# Protective Role of Carbonic Anhydrases III and VII in Cellular Defense Mechanisms upon Redox Unbalance

**DOI:** 10.1155/2018/2018306

**Published:** 2018-08-05

**Authors:** Anna Di Fiore, Daria M. Monti, Andrea Scaloni, Giuseppina De Simone, Simona M. Monti

**Affiliations:** ^1^Institute of Biostructures and Bioimaging, National Research Council, 80134 Naples, Italy; ^2^Department of Chemical Sciences, University of Naples Federico II, 80126 Naples, Italy; ^3^Proteomics and Mass Spectrometry Laboratory, ISPAAM, National Research Council, 80147 Naples, Italy

## Abstract

Under oxidative stress conditions, several constitutive cellular defense systems are activated, which involve both enzymatic systems and molecules with antioxidant properties such as glutathione and vitamins. In addition, proteins containing reactive sulfhydryl groups may eventually undergo reversible redox modifications whose products act as protective shields able to avoid further permanent molecular oxidative damage either in stressful conditions or under pathological circumstances. After the recovery of normal redox conditions, the reduced state of protein sulfhydryl groups is restored. In this context, carbonic anhydrases (CAs) III and VII, which are human metalloenzymes catalyzing the reversible hydration of carbon dioxide to bicarbonate and proton, have been identified to play an antioxidant role in cells where oxidative damage occurs. Both proteins are mainly localized in tissues characterized by a high rate of oxygen consumption, and contain on their molecular surface two reactive cysteine residues eventually undergoing S-glutathionylation. Here, we will provide an overview on the molecular and functional features of these proteins highlighting their implications into molecular processes occurring during oxidative stress conditions.

## 1. Introduction

In physiological conditions, reactive oxygen species (ROS) are generated intracellularly as a result of metabolism in peroxisomes, mitochondria, and by several cytosolic enzymes [1]. However, their generation is also triggered by exogenous sources, such as UV-light, chemotherapeutics, inflammatory cytokines, and ionizing radiations [1, 2]. Normally, the levels of ROS in cells are tightly regulated by sophisticated enzymatic and nonenzymatic antioxidant defense systems, such as catalase, superoxide dismutase, glutathione peroxidase, glutathione (GSH), and vitamins (vitamin A, C, and E). Generated ROS, within certain boundaries, are fundamental to preserve cell homeostasis and serve as important regulatory mechanisms for many cellular activities [1]. When ROS levels are unbalanced, detrimental effects on the physiological functions of the cell may occur. This can eventually lead to accelerated aging, age-related diseases, and, ultimately, to cell death [1]. ROS, which include oxygen-centered radical species, as hydroxyl radical (^**·**^OH), superoxide anion (O_2_^**·**-^), and peroxyl radical (R-O_2_^**·**^); and nonradical compounds, as hydrogen peroxide (H_2_O_2_), hypochlorous acid (HOCl), and ozone (O_3_), are known to modify proteins, nucleic acids, and lipids through a number of oxidative pathways [3–6].

In proteins, oxidative posttranslational modifications can either be permanent, as result of irreversible molecular oxidative damage, or temporary, which prevent lasting oxidative damage under stressful cellular conditions [7]. Cysteine is the most susceptible residue toward oxidative modifications. This is a nonabundant amino acid, being less than 3% compared to the other residues of the mammalian proteome [8]. It is nucleophilic, redox sensitive, and can undergo reversible and irreversible modifications in response to an altered localized redox environment [9]. Cysteine thiols have a pKa value of about 8.5 [10]. For this reason, they are not reactive at physiological pH values. However, within the protein three-dimensional structure, cysteines can be affected by the presence of specific residues altering their reactivity. In particular, the spatial proximity of acidic residues will raise the pKa value of thiol group, leaving cysteine uncharged and making it less prone to modification [11]. On the contrary, an alkaline environment given by the vicinal presence of basic residues can lower the corresponding pKa value, facilitating thiol deprotonation [11]. Cys modification is also affected by accessibility, since steric factors may prevent its alteration, even if the pKa of the thiol residue is relatively low [12]. For these reasons, oxidation of reactive cysteines is a highly selective process [13–15].

**Figure 1:**
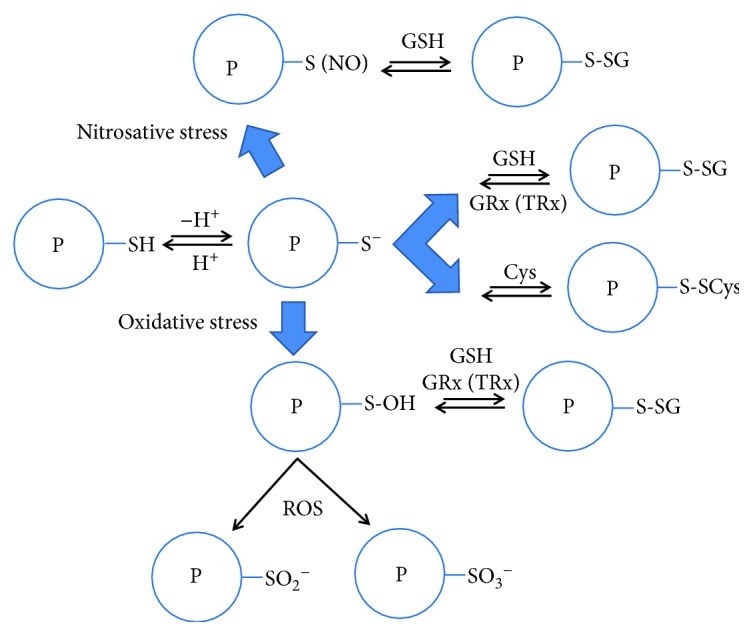
Schematic representation of protein oxidative modifications involving its reactive cysteine and nonprotein thiols with low molecular weight mass.

The thiolate anion renders proteins susceptible targets for a variety of oxidative modifications, generating cysteine derivatives containing intramolecular and intermolecular disulfide linkages, adducted species bearing disulfide bonds with low-molecular mass nonprotein thiols (mainly with GSH and to a lesser extent with free cysteine, forming PS-SG and PS-SCys, resp.) and sulfenic acid (P-SOH) (Figure 1). Sulfenic acid-containing proteins can undergo glutathionylation or be further oxidized leading to the irreversible formation of sulfinic (P-SO_2_H) and/or sulfonic acid (P-SO_3_H) derivatives [9]. The latter oxidative modifications often can irreversibly alter the structure and function of the involved protein. On the contrary, protein S-glutathionylation is a reversible reaction that can be efficiently reverted primarily by glutaredoxin (GRx) [16] and also by thioredoxin (TRx) [17], sulfiredoxin [18], or spontaneously in the presence of a high ratio of GSH/GSSG [19] (Figure 1). In oxidative stress conditions, protein thiolate anion may also undergo S-nitrosylation with reactive nitrosative species (RNS). Resulting protein S-nitrosothiol has a relatively short half-life since it reacts with physiological GSH forming the S-glutathionylated protein [9, 20, 21] (Figure 1).

Reversible S-modification can affect protein structure and function; thus, it has been reported as a molecular switch able to reversibly activate or deactivate regulatory processes, such as serving to transduce redox signals, control of gene expression, cell proliferation, and apoptosis [15, 22]. Besides its role in redox signal transduction, S-glutathionylation has been suggested also to be a cellular mechanism by which cells preserve enzyme functionality from further irreversible oxidation [22, 23].

Among the different antioxidant systems that cells have developed, it has been reported that proteins containing reactive cysteines could play a protective role against oxidative insult occurring either in stressful conditions or under pathological circumstances. These proteins participate in the cellular defense system by means of reversible thiol modification of their cysteine residues. In this view, we here report on the antioxidant role of two human metalloenzymes, namely, carbonic anhydrases (CAs) III and VII, whose function in mediating the oxidative insult and aging has been recently reported [24].

CAs are ubiquitous enzymes [25], encoded by seven genetically distinct gene families: *α*-, *β*-, *γ*-, *δ*-, *ζ*-, *η*-, and *θ*-CAs [25–30]. Human CAs (hCAs) belong to the *α*-class with fifteen isoforms being so far identified that differ in tissue distribution, catalytic activity, response to inhibitors, and cellular localization. Indeed, five isoforms are cytosolic (CAs I–III, VII, and XIII), four are membrane-associated (CAs IV, IX, XII, and XIV), two are mitochondrial (CAs VA and VB), and one is secreted in milk and saliva (CA VI) [25, 31, 32]. CAs catalyze a simple physiological reaction, the reversible hydration of carbon dioxide to bicarbonate and proton, following a two-step mechanism [33, 34]. Several studies reported that CAs are involved in a variety of physiological processes, such as acid-base balance, respiration, carbon dioxide and ion transport, bone resorption, and ureagenesis. Consequently, abnormal levels or activities of these enzymes have been often associated with different human pathological conditions [31, 35].

Among the cytosolic CA isoforms, CA III and CA VII are enzymes having properties that deserve further attention [24]. In particular, recent studies showed that these proteins are mainly present in tissues characterized by a high oxygen consumption rate, such as skeletal muscle, liver, and brain, where they could participate in cell defense processes counteracting oxidative damage [24, 36]. In this review, by examining CA III and CA VII functional and structural features, we will provide insights into their newly proposed protective role against oxidative stress.

### 1.1. Tissue Distribution, Catalytic Activity, and Molecular Features of CA III and CA VII

The distribution pattern of CA III has been investigated using different techniques, such as Western blotting and immunohistochemistry experiments, indicating that this isoform is highly expressed in liver and skeletal muscle [37] and at lower levels in other tissues [37–45]. CA VII was thought initially to have a more limited tissue distribution, being detected only in some brain regions including the cortex, hippocampus, and thalamus where it plays a role as a molecular switch for GABAergic excitation [46–48]. Subsequently, CA VII has been found also in other human tissues such as colon, muscle, and liver [49], revealing a strong similarity with CA III.

**Figure 2:**
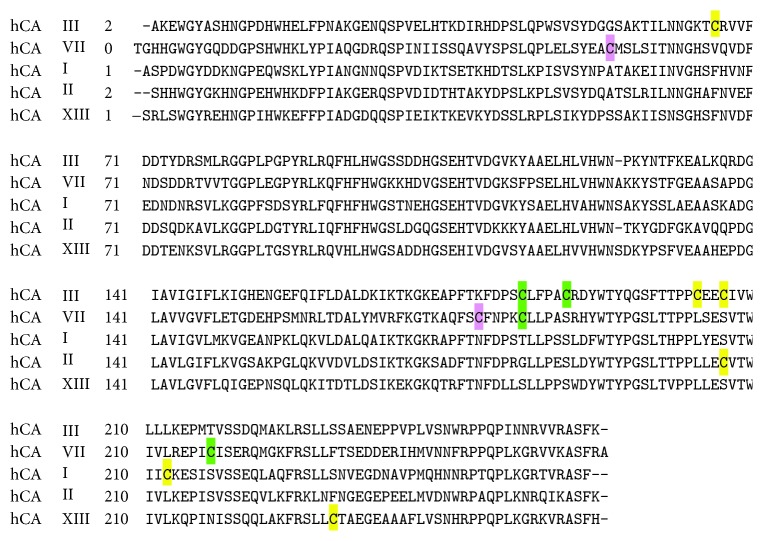
Sequence alignment of cytosolic hCAs. Reactive cysteines of CA III and VII are highlighted in green and reduced ones in yellow, whereas CA VII cysteines involved in the formation of an intramolecular disulfide bond are in pink.

CA III and CA VII present also high similarity in their primary structures with 49% sequence identity (Figure 2). Notably, they contain a higher number of cysteine residues (5 and 4, resp.) with respect to other cytosolic CAs having only a single cysteine (Figure 2) [24].

It has been reported that both enzymes have two highly reactive cysteines on the protein surface, namely, Cys183 and Cys188 for CA III and Cys183 and Cys217 for CA VII (numbering refers to hCA I isoform [50]). These cysteines have been reported to be S-glutathionylated both *in vitro* [51] and *in vivo* [52, 53], without affecting enzyme catalytic activity [51, 53]. Thus, the localization of CA III and CA VII in organs and tissues characterized by a high propensity for oxidative stress, combined with the presence of reactive sulfhydryls in their primary structure, provided the first evidence that these enzymes may act as oxyradical scavengers involved in cell protection from oxidative damage.

Despite the above-described similarities, CA III and CA VII present a very different catalytic efficiencies for hydration of CO_2_, with CA VII being one of the most efficient catalysts among mammalian isoforms [51, 54] and CA III being the worst one (k_cat_/K_M_ of 0.3 × 10^6^ M^−1^ s^−1^ for CA III and of 7.2 × 10^7^ M^−1^ s^−1^ for CA VII) [55].

Structural studies on hCA III [56] and on a mutated form of hCA VII, named dmCA VII [54, 57], showed that both enzymes adopt a three-dimensional arrangement similar to that of other cytosolic CAs [50, 58]. Both proteins are monomeric, and their fold presents a central ten-stranded *β*-sheet surrounded by additional *β*-strands and several *α*- and 3_10_-helices. Their active site is located in a large conical cavity, containing the catalytic zinc ion at the base, which is coordinated by three conserved histidine residues (His94, His96, and His119). The X-ray structures of bovine and rat CA III have also been solved [53, 59].

Remarkably, the structural analysis of rat CA III provided very interesting data clarifying the molecular determinants responsible for the above-mentioned high redox reactivity of Cys183 and Cys188. Indeed, these two residues, which are located on the molecular surface of the protein, were found S-glutathionylated in the crystal structure [53]. Since analysis of the binding sites gave no evidence for a specific recognition of GSH, it was suggested that S-glutathionylation was due to the high reactivity of Cys183 and Cys188 and to the great abundance of GSH in cell, which reaches *in vivo* millimolar concentration [2, 23, 60]. The disulfide linkage between the cysteine residue and the GSH molecule does not alter the overall structure of the protein, nor the conformation of residues located close to Cys183 and Cys188, in agreement with the observation that S-glutathionylation does not have effect on the catalytic activity of the enzyme [53]. Moreover, the electron density maps of the two Cys-GSH adducts indicated a conformational flexibility of the glutathionyl moieties, with the disulfide bridge involving Cys183 adopting two different orientations, and that involving Cys188 only one.

**Figure 3:**
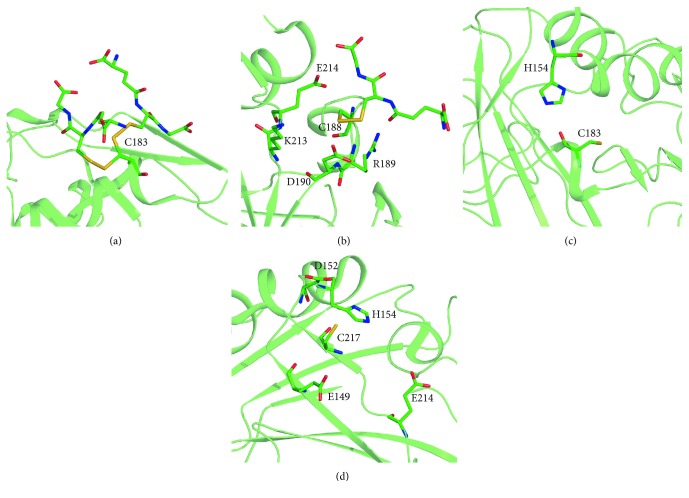
Chemical environment of CA III and VII reactive cysteines. (a) S-glutathionylated Cys183 of CA III showing two alternative conformations. (b) S-glutathionylated Cys188 of CA III surrounded by Arg189, Asp190, Lys213, and Glu214 which affect its pKa. (c) Model of Cys183 in CA VII structure adjacent to His154. (d) Model of Cys217 in CA VII structure showing the nearby acidic and basic residues.

Among the two cysteines, Cys188 is located in an environment characterized by a lower negative charge, thus explaining its greater propensity to react [61]. On the contrary, Cys183 is located in a depression of the surface showing a greater negative charge, making this residue less reactive. Two positively charged residues surrounding Cys188, namely, Lys213 and Arg189, were hypothesized to act as main modulators of thiol reactivity, lowering its pKa value. Interestingly, mutagenesis studies showed that only Lys213 is responsible for the lowering of the pKa value of Cys188, whereas Arg189 seems not to affect it. In addition, the acid residues Asp190 and Glu214 were also found to interact with the thiol of Cys188, decreasing its reactivity and partially counteracting the presence of the above-mentioned basic residues (Figures 3(a) and 3(b)) [61]. The crystallographic structure of S-glutathionylated CA III provided also information on the different reactivity of adducted protein thiols towards the process of deglutathionylation, with the Cys188-glutathione adduct being more accessible to nucleophilic attack compared to the corresponding Cys183-adduct, due to its greater steric accessibility [53].

In the case of CA VII, Cys54 and Cys178 were proved to be involved in an intramolecular disulfide bond, whereas the two remaining cysteines, Cys183 and Cys217, are exposed on the molecular surface [54, 57]. At present, no crystallographic information is available for S-glutathionylated Cys183 and Cys217. Thus, starting from the crystallographic structure of dmCA VII, we have built a model of hCA VII and used it to investigate the chemical environment potentially affecting Cys183 and Cys217 reactivity. Interestingly, we observed that Cys183 is located within a region devoid of acidic residues. The presence of a basic residue (His154) distant about 5 Å from the side chain of Cys183 could lower its pKa value, making it highly reactive (Figure 3(c)). His154 is located also in close proximity of Cys217; however, the presence of several acidic residues located nearby the thiol group may diminish its reactivity (Figure 3(d)). Further studies are necessary to clarify this matter.

### 1.2. Role of CA III as Antioxidant Agent

Important results elucidating the physiological role of CA III in aging and aging-related processes have been obtained from a study on the nucleus pulposus (NP) phenotype [62]. NP is part of the intervertebral disc, and its integrity is strictly related to intervertebral disc degeneration. This, in turn, is associated with low back and neck pain, leading to a very common disability in the United States [63, 64]. One of the major etiologic factors responsible for this pathological condition is aging disc [65], which is characterized by the time-dependent accumulation of cellular and molecular damage, predominantly caused by oxidative and inflammatory processes [65, 66]. Notably, NP is a hypoxic niche where the two hypoxia-inducible CAs IX and XII are robustly expressed and regulate intracellular pH level, which is essential for the maintenance of cellular functions [67]. mRNA and protein expression of CA III are hypoxia-sensitive, being upregulated in low oxygen tension. However, unlike CAs IX and XII, CA III expression is insensitive to HIF-1*α*, and this isoform does not play a role in the regulation of intracellular pH homeostasis [62], but it participates to the antioxidant defense mechanism of NP cells. Indeed, NP cells silenced for CA III expression showed high sensitivity to oxidative stress-dependent apoptosis through caspase-3 activation. Therefore, it has been suggested that mechanisms regulating CA III expression may represent novel therapeutic targets to reduce the negative effects of oxidative damage associated with aging in the degeneration of the intervertebral disc [62].

In agreement with this proposed protective role of CA III, it was demonstrated that this protein inhibited apoptosis in H_2_O_2_-stressed mature osteocytes [68] and in cotransfected NIH/3T3 cells [69]. In particular, CA III expression increased with osteoblast differentiation and was also involved in diminishing ROS production and in protecting cells from hypoxic stress [68]. Interesting data were also reported on the repression of CA III gene transcription in Rat1 cells expressing high level of Evi1 [70], a zinc finger protein involved into cancer progression [71]. In these cells, Evi1 overexpression corresponded to a decreased level of CA III and to an enhanced sensitivity to H_2_O_2_-induced apoptosis [70]. These results may be used to exploit novel therapies targeting oxidative stress.

Even though many papers describe the role of CA III in cellular defense processes from oxidative insult, very little information is available on the molecular mechanisms responsible for this behavior. It has been suggested that the antioxidant activity of this protein is related to the presence of the above-described reactive cysteines, which *in vivo* are S-glutathionylated [53]. To get more insights into this process, Mallis and coworkers investigated in detail the chemical modification of CA III sulfhydryl groups upon exposure to different oxidant agents, such as H_2_O_2_, HOCl, or peroxy radicals [72]. The authors revealed that the type of cysteine modification depends on cellular GSH content. Indeed, CA III was reversibly S-glutathionylated when GSH concentration was approximately equimolar to that of protein thiols, whereas irreversible oxidation of cysteines to sulfinic or sulfonic acid derivatives occurred at low GSH levels. In agreement with these data, CA III was found to be highly and irreversibly oxidized in old rats with respect to young animals, since reduced GSH levels are a hallmark of aging.

Further insights into the role and mechanism of action of CA III as antioxidant agent were obtained by Zimmerman et al. through the evaluation of S-glutathionylation and irreversible oxidation of the two protein reactive cysteines in the presence of increasing intensities of the oxidative stress insult [73]. These analyses indicated that Cys183 and Cys188 were differentially oxidized in skeletal muscle when different oxidative stress conditions were induced. Specifically, only Cys188 underwent reversible S-glutathionylation under a mild or brief redox stress, involving about 20% of total skeletal muscle protein, whereas a prolonged or harsh stress produced irreversible oxidation of both sulfhydryl groups. Thus, the high content of CA III in skeletal muscle could represent a storage of reactive sulfhydryl moieties able to repair acute and chronic oxidative insults. Moreover, in resting skeletal muscle, less than 10% of CA III was S-glutathionylated, suggesting that cysteine oxidation may represent a regulative physiological mechanism performed by the enzyme within the cell. A further proof of a possible involvement of CA III in the glutathione-mediated antioxidant processes was obtained by comparing the gene expression profile of CA3-knockout and wild type mice by microarray strategy [73]. Even though CA III-deficient mice had a normal development, fertility, and longevity, at least under experimental standard laboratory conditions, they showed a transcriptional alteration of more than 500 out of 12,000 genes analyzed; most of them were associated with the GSH-mediated antioxidative system [74].

### 1.3. Role of CA VII as Antioxidant Agent

Available data on reactive cysteines in CA VII [51], together with the observation that this enzyme is expressed in tissues with a high oxygen consumption rate (similarly to CA III), led us to hypothesize that this protein may have a functional role *in vivo*, that is of protecting cells from oxidative stress [52]. To test this hypothesis, human cancer (HeLa) cells, which usually do not express endogenous CA VII, were transiently transfected to express the wild-type protein, in the presence or in the absence of oxidative stress. Consistent with our hypothesis, cells expressing CA VII were less sensitive to apoptosis induced by oxidative stress. This was clearly demonstrated by measuring: (i) apoptotic protein levels and (ii) apoptotic cells. In particular, mock-transfected cells showed a significant alteration in procaspases (8 and 3), Bcl-2 and Bax levels after induction of oxidative stress, whereas corresponding protein levels were almost unaltered in CA VII expressing cells. Moreover, in the presence of oxidative stress, an increase in CA VII expression was observed, suggesting an attempt by the cell to protect itself by overexpressing the protein [52]. A further confirmation of the importance of the cysteine residues was obtained by performing the same experiments in the presence of a mutated version of the protein, in which all cysteines were replaced by serines (TM-CA VII). When cells were transiently transfected with the vector encoding for the mutated protein and then stressed, no protection was observed. Indeed, a similar alteration in procaspase-3 and 8, Bax and Bcl-2 levels as well as apoptotic cells, was observed for mock and TM-CA VII transfected cells [52]. Thus, cysteine residues in CA VII exert a protective functional role. Notably, the lack of the protective effect was not related to a different catalytic activity of the mutated enzyme, since it showed the same kinetic parameters of native CA VII [52].

It is commonly accepted that increased levels of ROS are related to oxidative stress, and may lead to the development of many pathologies, including cancer [75–78]. Interestingly, a reduced CA VII expression is observed in colorectal carcinoma [79], thus allowing one to speculate that low protein levels may be related to a higher cellular sensitivity to oxidative stress and cancer progression.

## 2. Conclusions

In summary, cells have developed different sophisticated defense systems to counteract oxidative stress, and their ability to respond to ROS/RNS production is connected to aging, cancer, and other disease states. Among the several molecular systems, the contribution of CA III and CA VII as scavengers towards oxidative insult has been recently proposed, highlighting an unexpected functional role of these two CAs. Notably, these CAs are abundantly expressed in tissues such as brain, liver, and skeletal muscle, which present high oxygen consumption rates. By means of different approaches including *in vitro* and *in vivo* experiments, it was shown that CA III and CA VII exert their protective role due to the presence of reactive cysteines on their protein surface.

In particular, from a molecular point of view, the chemical environment generated by residues nearby the reactive sulfhydryls of CA III and CA VII assists the formation of a thiolate anion which may undergo S-glutathionylation. This reversible modification is thought to protect cellular proteins and preserves their functionality under oxidative stress conditions.

Significant findings on the peculiar role of these proteins were obtained by the observation that both CA III and CA VII protect cells from apoptosis induced by oxidative agents, thus participating in the cellular defense system. Further studies are necessary to better clarify their molecular and biological mechanism and a putative role as novel antiaging molecules.
